# *Fusarium graminearum:* pathogen or endophyte of North American grasses?

**DOI:** 10.1111/nph.14894

**Published:** 2017-11-21

**Authors:** Lotus A. Lofgren, Nicholas R. LeBlanc, Amanda K. Certano, Jonny Nachtigall, Kathryn M. LaBine, Jakob Riddle, Karen Broz, Yanhong Dong, Bianca Bethan, Christopher W. Kafer, H. Corby Kistler

**Affiliations:** ^1^ Department of Plant and Microbial Biology University of Minnesota St Paul MN 55108 USA; ^2^ Department of Plant Pathology University of Minnesota St Paul MN 55108 USA; ^3^ Metanomics GmbH RBP/HA ‐ Tegeler Weg 33 10589 Berlin Germany; ^4^ Cereal Disease Laboratory USDA ARS St Paul MN 55108 USA; ^5^ BASF Plant Science LP 26 Davis Dr. Research Triangle Park NC 27709 USA

**Keywords:** co‐evolution, endophyte, *Fusarium graminearum*, mycotoxin, native grasses, trichothecene

## Abstract

- Mycotoxin‐producing *Fusarium graminearum* and related species cause Fusarium head blight on cultivated grasses, such as wheat and barley. However, these *Fusarium* species may have had a longer evolutionary history with North American grasses than with cultivated crops and may interact with the ancestral hosts in ways which are biochemically distinct.
- We assayed 25 species of asymptomatic native grasses for the presence of *Fusarium* species and confirmed infected grasses as hosts using re‐inoculation tests. We examined seed from native grasses for the presence of mycotoxin‐producing *Fusarium* species and evaluated the ability of these fungi to produce mycotoxins in both native grass and wheat hosts using biochemical analysis.
- Mycotoxin‐producing *Fusarium* species were shown to be prevalent in phylogenetically diverse native grasses, colonizing multiple tissue types, including seeds, leaves and inflorescence structures. Artificially inoculated grasses accumulated trichothecenes to a much lesser extent than wheat, and naturally infected grasses showed little to no accumulation.
- Native North American grasses are commonly inhabited by *Fusarium* species, but appear to accommodate these toxigenic fungi differently from cultivated crops. This finding highlights how host identity and evolutionary history may influence the outcome of plant–fungal interactions and may inform future efforts in crop improvement.

## Abstract 2

See also the Commentary on this article by Selosse *et al.*, **217**: 968–972.

## Introduction

With the growing use of directed metagenomics to characterize the microbes associated with plants, it has become increasingly common to consider a wider view of fungal endophytes. Studies on fungal endophytes of grasses have been largely influenced by the important symbiotic fungi in the genus *Epichloë* and related Clavicipitaceae. *Epichloë* species produce ergot alkaloids that render their host resistant to herbivore pressure (Florea *et al*., 2017). These fungi are often obligate endophytes which co‐evolved with their host species, and are known to establish multigenerational associations with their host by way of vertical (i.e. seed) transmission (Saikkonen *et al*., 2016). Although these mutualistic, co‐evolved fungi form the type of associations classically regarded as endophytic, it is becoming clear that endophytic associations encompass fungal species that employ a diverse spectrum of ecological strategies (Rodriguez *et al*., 2009).

Fungi of the endophytobiome may be free living, non‐systemic, horizontally transferred by way of primary infection and/or may provide no obvious benefit to the host (Sánchez Márquez *et al*., 2012). The outcome of these endophytic relationships may be host species dependent, with the same fungal species taking on multiple roles depending on host association (van Kan *et al*., 2014). The products of these interactions probably depend on the evolutionary history between the plant and fungal partner. There is evidence that endophytic fungi may exhibit some level of host adaptation, as the same fungal species have been isolated from the same host over time and geographic space. For example, *Fusarium avenaceum* and *F. tricinctum* have repeatedly been reported as symptomless colonizers of wild grass species (Inch & Gilbert, 2003; Harrow *et al*., 2010; Turkington *et al*., 2011; Postic *et al*., 2012) and occasionally forbs (Hodgson *et al*., 2014). *Fusarium verticillioides* has been reported as both a symptomless endophyte and a pathogen of corn (Bacon & Hinton, 1996). Likewise, members of the *Fusarium graminearum* species complex (FGSC) have also been recovered from several non‐symptomatic wild grass species (Inch & Gilbert, 2003; Goswami & Kistler, 2004; Starkey *et al*., 2007; Varga *et al*., 2015).

Fusarium head blight (FHB) disease of wheat and barley is among the most important diseases of cereal crops worldwide. The disease not only causes serious yield reductions, but also contaminates the grain with harmful trichothecene mycotoxins, rendering it unacceptable for food or feed. FHB is caused predominantly by fungi within the FGSC. Numerous biogeographically structured lineages of the FHB pathogen have now been discovered (Starkey *et al*., 2007). The name *F. graminearum sensu stricto* (*s.s*.) has been retained for the predominant lineage in North America and, although this species is found worldwide, evidence suggests that it may have arisen in North America (Aoki *et al*., 2012). Surveys of FHB pathogens have uncovered two apparent sister taxa of *F. graminearum s.s*. endemic to North America, *F. gerlachii* and *F. louisianense*, which also belong to the FGSC (Starkey *et al*., 2007; Gale *et al*., 2011; Sarver *et al*., 2011).

Fusarium head blight pathogen surveys of the USA have uncovered an unexpected diversity of trichothecene mycotoxins. In addition to strains that produce the common trichothecenes 15‐acetyl,deoxynivalenol (15ADON) and 3‐acetyl,deoxynivalenol (3ADON), others have been shown to produce nivalenol (NIV) (Gale *et al*., 2011) or the newly described trichothecene toxin NX‐2 (Liang *et al*., 2014; Varga *et al*., 2015). Notably, *F. gerlachii* and NX‐2‐producing strains of *F. graminearum s.s*. were first discovered growing on symptomless wild grasses in Minnesota, USA (Starkey *et al*., 2007; Varga *et al*., 2015), and were only later discovered as FHB pathogens on wheat. This, coupled with evidence that *F. graminearum s.s*. probably arose as a North American species, suggest that this lineage may have arisen separately from its generally recognized hosts, wheat and barley, which it would have first encountered only when these crops were introduced to North America during the last 400 yr.

Instead, *F. graminearum s.s*. may have evolved as a non‐pathogenic endophyte of North American grass species. Endophytes are generally recognized as the subsurface microbiome of plants, although other definitions have also been offered (Hyde & Soytong, 2008). Early inoculation studies using the FHB pathogen suggested that its host range encompassed non‐cultivated grasses (Purss, 1969), including North American wild rye species *Elymus canadensis* and *E. virginicus* (MacInnes & Fogelman, 1923). A more recent survey of non‐inoculated, non‐symptomatic native grasses of North America identified these hosts as potential reservoirs of inoculum of the FHB pathogen (Inch & Gilbert, 2003). However, given the likelihood that *F. graminearum s.s*. arose in North America, the symptomless interactions with native grasses possibly represent the natural niche of the fungus in association with a co‐adapted host. This view of the pathogen may represent a new paradigm for understanding how certain phytopathogenic interactions arise. Rather than having co‐evolved in an arms race‐driven interaction, FHB may instead be a maladaptive response between FGSC and plants that arose on different continents brought together by human activity only very recently in evolutionary time.

The purpose of this study was to examine the host range for members of the FGSC in the state of Minnesota by sampling symptomless native grasses for the presence of *Fusarium*. Plant species harboring FGSC fungi were confirmed as hosts by re‐inoculation tests conducted in the glasshouse. Seed obtained from native grasses was also examined for the presence of FGSC strains. Because disease symptoms on cultivated wheat are dependent on the ability of the fungus to produce trichothecene mycotoxins (Proctor *et al*., 1995; Goswami & Kistler, 2005; Jansen *et al*., 2005), naturally infected tissue and inoculated native grasses were tested for the presence of these and other mycotoxins. Of interest was whether trichothecenes accumulate during symptomless interactions of FGSC strains with native grasses and/or whether these grasses may have evolved to cope with the toxic effect of accumulated trichothecenes.

## Materials and Methods

### Sample collection from native grasses

**Figure 1:**
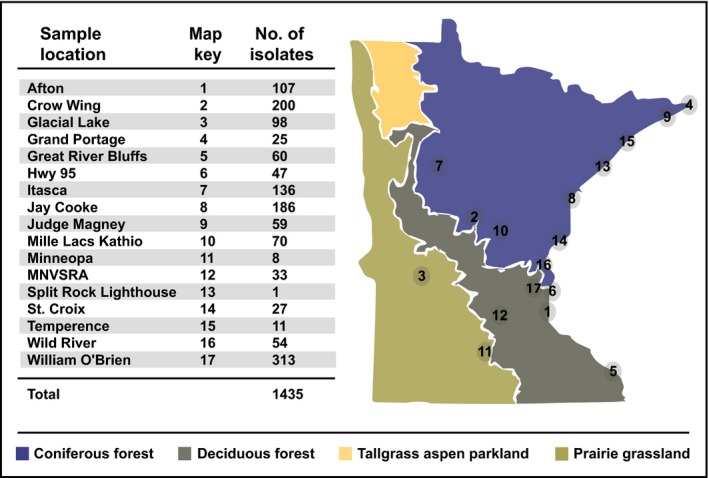
Locations of plant collections in the state of Minnesota (USA). Major vegetation zones are indicated by different colors. All locations are State Parks, except for Hwy 95 (State Highway 95) which parallels the St Croix River that forms the eastern border of much of the state. Numbers indicate the quantity of single‐spore cultures obtained from plant collections at that site.

Collections of 25 grass species were made from 22 June to 15 September 2015 (Supporting Information Table S1). All but one (*Phalaris arundinacea* – reed canarygrass) is considered to be native to North America (USDA Natural Resource Conservation Service – http://plants.usda.gov/java/). In an attempt to maximize the recovery of *Fusarium* in native plant interactions, sampling was targeted away from agricultural settings in Minnesota State Parks (special permit #201501) and along highways maintained by the Minnesota Department of Transportation (permit #US‐14‐65157) (Fig. 1). Flag leaves and the inflorescences of 348 individual plants taken across 17 sites were cut, GPS coordinates were taken for the collection site and plant tissue was kept on ice in the field and later refrigerated in the laboratory before processing. Individual leaves and flowers were divided in half, with one half used to obtain fungal cultures, whereas the other half was stored at −70 °C until mycotoxin analysis (see below – ‘Determination of mycotoxin content of native grasses’).

To isolate *Fusarium* cultures, plant tissue samples were wrapped in cheesecloth and sterilized in 70% ethanol for 2 min, followed by 0.825% sodium hypochlorite plus 0.1% Triton‐X‐100 for 2 min and then 95% ethanol for 30 s under sterile conditions, as described previously for the isolation of endophytic fungi (Higgins *et al*., 2014). Sterilized plant material was cut into 1–2‐cm pieces and plated on Nash–Snyder medium containing pentachloronitrobenzene (PCNB), streptomycin and neomycin (LeBlanc, 2015). This medium inhibits bacteria and many non‐*Fusarium* fungal species. Plated cultures were incubated for 1–2 wk until fungal colonies were large enough to be isolated and plated onto half‐strength potato dextrose agar (PDA; 12 g potato dextrose broth (Carolina Biological Supply Co., Burlington, NC, USA) + 15 g agar l^−1^). Each culture was then single‐spored onto either half‐strength PDA or, if fungal growth was poor, onto V8 medium (200 ml V8 juice, 2 g CaCO_3_ and 15 g Difco Bacto agar l^−1^) in order to obtain a pure culture. Strains were designated as NN (host designation given in Table S1) XX (the numbered isolate from that host). For example, strain EC10 was the tenth strain isolated from *E. canadensis*.

To test for the presence of *F. graminearum* in seed of native grasses, seed lots of 26 native grass species were acquired from Agricol (Evansville, WI, USA), World Seed Supply (Mastic Beach, NY, USA) or Prairie Restoration (Princeton, MN, USA). Seed from commercial sources, rather than naturally sourced seed, was used because of the difficulty in collecting a sufficient number of mature seeds for all grass species used in the study, as different species of plant exhibit differing rates and times for seed maturation. One hundred and twenty seeds per species were surface sterilized, plated separately onto Nash–Snyder medium amended with antibiotics, incubated for a maximum of 14 d and single‐spore isolated to ensure colony purity. None of the seed stock used in the study possessed signs associated with FHB (pink or bleached coloration, shrunken or wrinkled seed, etc.) and appeared to be viable regardless of infection status, as most species did not require vernalization and germinated on the agar medium during fungal isolation. Seed surface sterilization was confirmed by streaking representative seeds of each species across half‐strength PDA plates not amended with antibiotics. Plates showing an absence of fungal or bacterial growth were considered to represent seeds that had been successfully surface sterilized. Where fungal or bacterial growth was present, the sterilization time was increased by 30 s for that species and the assay was repeated until a clean control was obtained.

### Taxonomic assignment of fungal cultures

DNA was extracted from pure cultures in accordance with the Sigma REDExtract‐N‐Amp DNA extraction protocol (http://www.sigmaaldrich.com/technical-documents/protocols/biology/redextract-n-amp.html). The RNA polymerase II (RPB2) gene was used for strain identification by sequencing the PCR‐amplified gene using primers RPB2‐5F2 (5′‐GGGGWGAYCAGAAGAAGGC‐3′) and RPB2‐7CR (5′‐CCCATRGCTTGYTTRCCCAT‐3′), resulting in an amplicon size of *c*. 1.2 kb. PCR was performed in 20‐μl reactions with 10 μl REDExtract‐N‐Amp™ PCR ReadyMix™ (Sigma‐Aldrich, St Louis, MO, USA), 7 μl nuclease‐free H_2_O, 10 pmol of each primer and 1.5 μl of DNA template. DNA amplification was conducted in an Eppendorf thermocycler with the following program: initial denaturing at 95°C for 5 min, followed by 35 cycles of denaturing at 95°C for 1 min, annealing at 57°C for 1 min, extension at 72°C for 1 min and a final annealing at 72°C for 10 min. The PCR product (5 μl) was run on a 1% agarose gel containing either SYBRsafe (Thermo Fisher Scientific Inc., Waltham, MA, USA) or Greenview (GeneCopoeia Inc., Rockville, MD, USA) with 0.5 × TBE buffer for gel electrophoresis. The RPB2 PCR product, together with a 1‐kb ladder, used as a size standard, was visualized using ultraviolet light. Based on the gel electrophoresis results, samples were cleaned using 9 μl ExoSAP‐IT^®^ PCR Product Cleanup (Thermo Fisher) diluted to a 1 : 3 dilution with nuclease‐free H_2_O added to 15 μl of the remaining PCR product. The samples were cleaned by incubation at 37°C for 45 min and 85°C for 15 min to inactivate ExoSAP‐IT^®^ enzymes. The cleaned samples with the brightest and weakest fluorescence from each gel were quantified using a Hoefer fluorometer. All samples contained *c*. 3–15 ng μl^−1^ of DNA and were Sanger sequenced at the University of Minnesota Genomics Center. The resulting sequences were trimmed in 4Peaks v.1.8 (http://nucleobytes.com/4peaks).

For initial taxonomic identification of cultures, single‐pass RPB2 sequences were aligned to an in‐house RPB2 sequence database (LeBlanc *et al*., 2017) using Megablast and Tblastx v.2.5.0+ (Altschul *et al*., 1997; Camacho *et al*., 2009). Alignments to reference RPB2 sequences were evaluated based on bit‐score. Sequences aligning to non‐*Fusarium* references (e.g. *Microdochium nivale*) or displaying low protein alignment scores (bit‐score ≤ 500) were culled. Of the remaining 1000 sequences, 144 contained elements (e.g. internal stop codons) that prevented their submission to GenBank. As these data showed high levels of homology to reference RPB2 data and were classified to the same taxa as the remaining 856 sequences submitted to GenBank (accession nos MF43317 to MF434032), they were included in further analyses.

The 1000 RPB2 sequences were aligned using Mafft v.7 (Katoh & Standley, 2013) and then aligned to a reference RPB2 alignment (O'Donnell *et al*., 2013; Treebase accession: S12813) using Muscle (Edgar, 2004). The Evolutionary Placement Algorithm (Berger *et al*., 2011), as implemented in RaxML v.8.2.9 (Stamatakis, 2006), was used to place the original 1000 RPB2 sequences within the reference phylogeny published by O'Donnell *et al*. (2013), reconstructed as described in LeBlanc *et al*. (2017).

### Determination of mycotoxin content of native grasses

#### LC‐MS/MS

Methanol and acetonitrile (both LC/MS grade), acetic acid and ammonium acetate were purchased from Sigma Aldrich (Seelze, Germany); water was purified successively by reverse osmosis and a Milli‐Q plus system from Millipore (Merck Millipore, Darmstadt, Germany). Most mycotoxin standards were purchased from Sigma Aldrich and dissolved in acetonitrile. NX‐2 and NX‐3 (200 μg each) were obtained from Elisabeth Varga, IFA Tulln, Austria. The following compounds were used as internal standards (ISTDs): deepoxy‐deoxynivalenol, (U‐^13^C) Fumonisin B1 and (U‐^13^C) zearalenone. Up to 20 mg of the lyophilized sample material, 450 μl extraction solvent (acetonitrile with 1% formic acid) and 50 μl ISTD mixture (Qiagen, Hilden, Germany) were added together and extracted with 3‐mm steel balls for 10 min at 20 Hz using a bead mill (Retsch, Haan, Germany). The sample was centrifuged for 3 min at 1252 ***g*** and 300 μl of the supernatant were filtered through a 0.2‐μm filter (AcroPrep PTFE, Pall, Crailsheim, Germany) and subjected to UPLC/MS analysis. Detection and quantification were performed using an API 4000 LC‐MS/MS system (Sciex, Darmstadt, Germany) coupled to an Acquity UPLC system (Waters, Waldbronn, Germany). Chromatographic separation was performed on a reversed phase column (C_18_ material, Thermo Fisher, Dreieich, Germany) by gradient elution with water and methanol at 70°C. Both eluents contained 1% acetic acid and 0.1 mM ammonium acetate. MS detection was carried out using electrospray ionization in scheduled Multiple Reaction Monitoring (sMRM) mode.

#### GC‐MS

Each sample was weighed and placed into a 1‐dram glass vial capped with a screw cap and extracted by soaking and shaking with 2 ml of acetonitrile/water (84 : 16, v/v) for 24 h. The extract was passed through a mini‐column packed with C18 and aluminum oxide. One and a half milliliters of the filtrate were placed into a ½‐dram glass vial and evaporated to dryness under nitrogen. Twenty‐five microliters of TMS reagent (TMSI/TMCS = 100/1) were added, and the vial was rotated so that the reagent contacted with all residue in the vial. The vial was placed on a shaker for 10 min, and then 200 μl of isooctane were added, followed by 200 μl of HPLC water to quench the reaction. After vortexing, the upper layer was transferred to a GC vial. Selected ion monitoring (SIM) was used for GC‐MS analysis (Shimadzu GCMS‐QP2010; Shimadzu Corp., Kyoto, Japan).

### Inoculation of native grasses and Koch's postulates

Seeds of *E. canadensis*,* E. villosus*,* E. virginicus*,* Bromus latiglumis* and *Grevillea striata* were obtained from Prairie Restoration (http://www.prairieresto.com/) or Agrecol (http://www.agrecol.com/). Plants were grown from seed or commercially obtained as seedlings and grown in growth chambers or glasshouses at *c*. 20 ± 3°C with 16 h of daily supplemental light until anthesis. Plants were watered daily and treated with Nutricote 13‐13‐13 micronutrients, Type 100 (Plantco Inc., Brampton, ON, USA). At least eight spikelets were inoculated per replication using *Fusarium* strains derived from wild grass species (EC256 from *E. canadensis*, Evill18 from *E. villosus*, EV127 from *E. virginicus* or HJ15 from *Hordeum jubatum* – with HJ15 inoculated onto *H. jubatum* only) or an authentic FHB pathogenic strain (NRRL 31084) as a control. Inoculation of wheat and *Elymus* species was conducted using 10^4^ conidia in a 10‐μl volume as described previously (Goswami & Kistler, 2005). Single central spikelets within racemes or panicles of *B. latiglumis* and *G. striata*, respectively, were tagged and similarly inoculated with 10^4^ conidia. To investigate whether grass‐derived strains were similarly infective on wheat, *Triticum aestivum* var. Norm was inoculated with strains derived from *Panicum virgatum* (PV63, PV65, PV66) or *Elymus* species (Evill18, EV127, EC256), together with the control strain PH‐1. To test for natural infestation by *Fusarium* species, wild grasses were mock inoculated using the solution employed to suspend spores (sterile Millipore water + 1% v/v Triton X‐100). After inoculation, plants were placed in a humidity chamber for 2 d, and then transferred to a growth chamber. Fourteen days after inoculation, plants were prepared for mycotoxin analysis or Koch's postulate. Inoculated spikelets were assessed for the presence of the mycotoxins deoxynivalenol (DON), 3ADON, 15ADON, NIV or NX‐2 by GC‐MS, as described previously (Goswami & Kistler, 2005; Varga *et al*., 2015). For Koch's postulates, intact or disarticulated inflorescences were sterilized in 0.825% sodium hypochlorite plus 0.1% Triton‐X‐100 for 5 min, rinsed with sterile water and then blotted dry with sterile paper towels. Plant tissue was then plated onto agar containing PCNB, neomycin (6 ml l^−1^) and streptomycin (10 ml l^−1^). The assessment of *Fusarium* growth and spread within the plant was performed between 5 and 10 d after inoculation.

## Results

### Survey of native grasses

**Figure 2:**
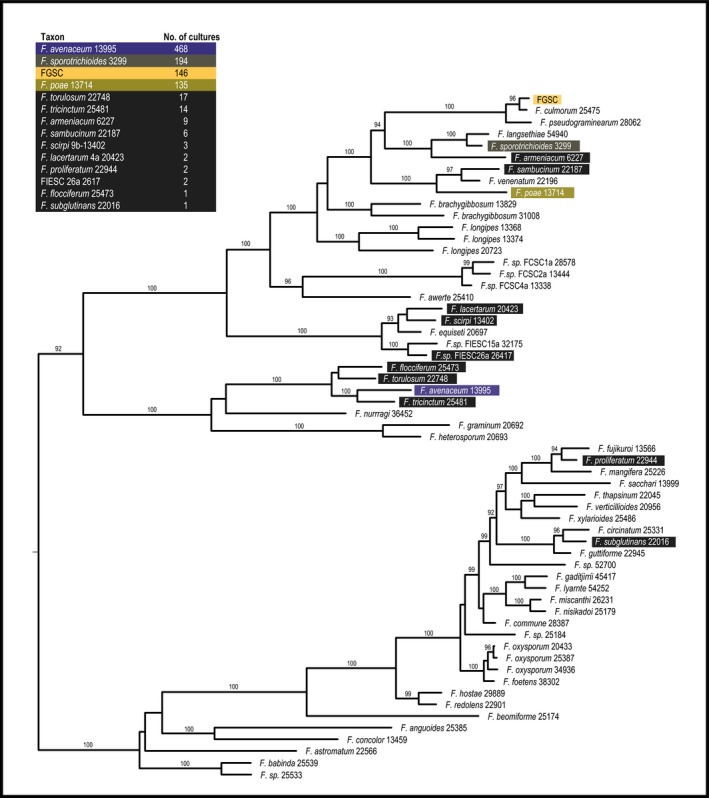
Placement of *Fusarium* taxa within a genus‐wide reference phylogeny using RPB2 sequences from culture. Individual RPB2 sequences from *Fusarium* cultures were placed within the genus‐wide *Fusarium* reference phylogeny (O'Donnell *et al*., 2013) using the Evolutionary Placement Algorithm (Berger *et al*., 2011). Taxa are ranked at the left according to abundance. The phylogeny displayed is a subset of the complete *Fusarium* phylogeny, displaying taxa relevant to the classified RPB2 sequences. *Fusarium graminearum* species complex (FGSC) isolates have been collapsed into a single node. Bootstrap values of 90 and above are displayed.

Of the 348 plant collections made throughout the state, 210 (60.3%) contained what appeared to be *Fusarium* based on morphological criteria; 1435 cultures were established (Table S1), and DNA was successfully extracted and amplified from 1096 single‐spore isolates. From these sequences, 96 were excluded as a result of sequence quality or poor alignment to *Fusarium* species. Sequencing repeatedly identified a relatively small number of *Fusarium* spp. rather than a random assortment of species from across the *Fusarium* phylogeny (Fig. 2). Of the 1000 sequences remaining, most aligned with *Fusarium* species in the *F. tricinctum* clade, including *F. avenaceum* (468) and *F. tricinctum* (14), or in the *F. sambucinum* clade, including members of the FGSC (146), *F. sporotrichioides* (194) and *F. poae* (135).

**Figure 3:**
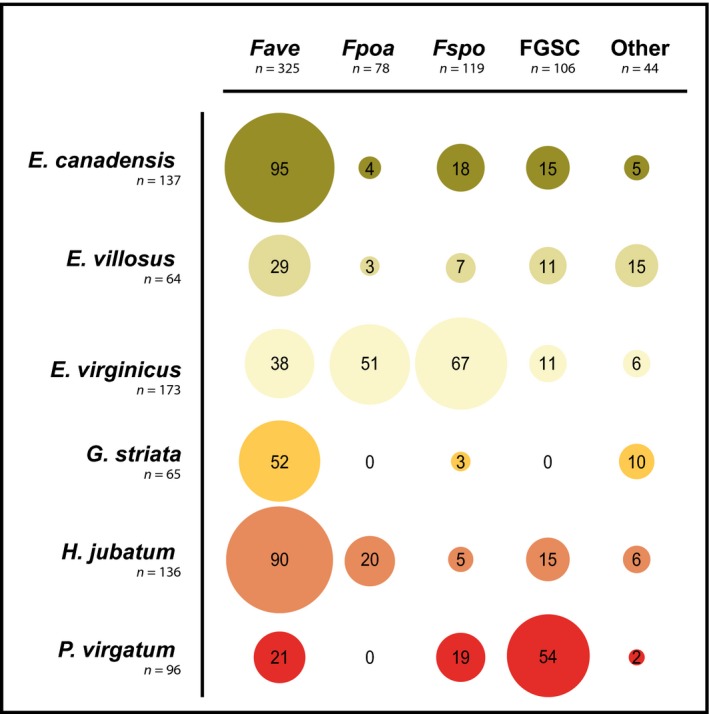
*Fusarium* isolated from six grass species. Approximately two‐thirds of *Fusarium* cultures were isolated from six grass species (*Elymus canadensis*,* E. villosus*,* E. virginicus*,* Glyceria striata*,* Hordeum jubatum*,* Panicum virgatum*). *Fave*,* F*.* avenaceum*;* Fpoa*,* F*.* poae*;* Fspo*,* F*. *sporotrichioides*; FGSC,* F*. *graminearum* species complex.

**Figure 4:**
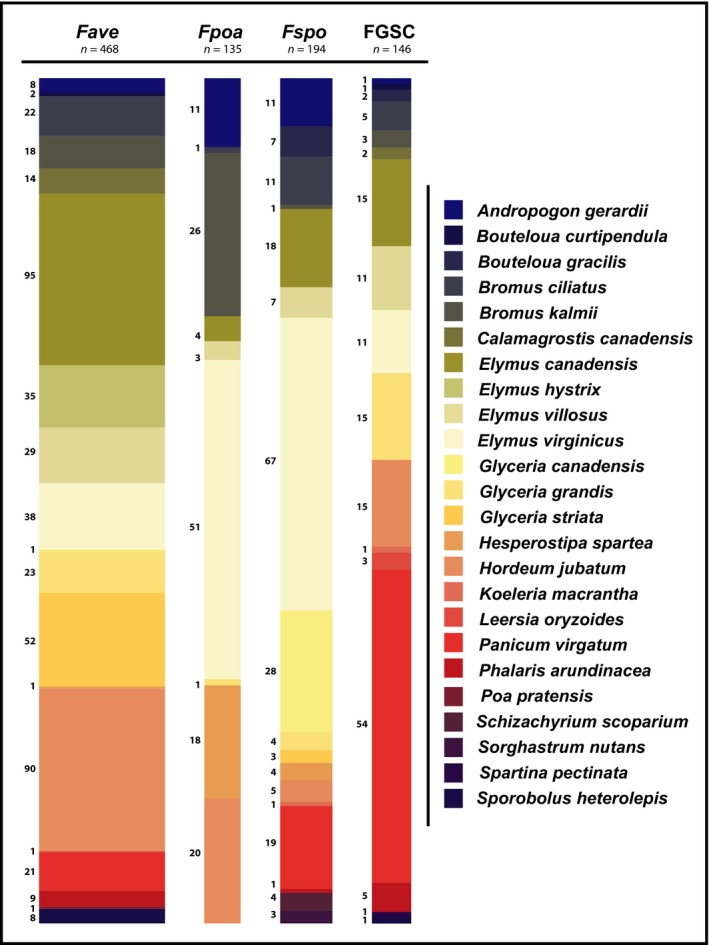
Plant host distribution of the four most commonly isolated *Fusarium* species. *Fave*,* F*.* avenaceum*;* Fpoa*,* F*.* poae*;* Fspo*,* F*. *sporotrichioides*; FGSC,* F*. *graminearum* species complex. The numbers adjacent to the spine plots represent the total number of cultures isolated from each plant species for the fungal species represented in each spine.

Approximately two‐thirds of the verified *Fusarium* isolates (671/1000) were from six grass species (Fig. 3). The most abundant *Fusarium* on four of these grass species (*E. canadensis*,* E. villosus*,* G. striata* and *H. jubatum*) was *F. avenaceum*. For the other two grass species, FGSC strains were most frequently identified from *P. virgatum*, whereas *F. sporotrichioides* was most commonly isolated from *E. virginicus*. However, each of these grass species also contained four or more *Fusarium* species in total. Each of the four most abundant *Fusarium* species were cultured from multiple plant species (Fig. 4). Members of the FGSC were found in 17 of the 25 grass species surveyed.

### Chemotypes of FGSC strains from wild grass species

The 146 FGSC strains were genotyped to determine what types of trichothecene toxin they would produce. All four trichothecene chemotypes were recovered at the following frequencies: 15ADON (82%), 3ADON (10%), NX‐2 (7%) and NIV (1%). Both FGSC NIV isolates were isolated from *Bromus ciliatus*, but at separate locations (Itasca and MNVSRA), and all 10 NX‐2 isolates were obtained from *G. grandis* at one location (Itasca).

### Mycotoxin content of plants naturally infected with *F. graminearum*

Forty‐four of the 145 samples (30%) showed detectable levels of mycotoxins characteristic of the FGSC (Table S2). Trichothecene mycotoxin accumulation was detected in nine plant species: *Andropogon gerardii*,* Bromus kalmia*,* Hesperostipa spartea*,* Hordeum jubatum*,* Koeleria macrantha*,* Leersia oryzoides*,* Panicum virgatum*,* Phalaris arundinacea* and *Schizachyrium scoparium*. Notably, although roughly one‐third of all plant samples analyzed were obtained from *Elymus* species (48 of 145), none of these contained detectable levels of trichothecene or any other mycotoxin. This is in sharp contrast with other plant species where 45% (44 of 97) of the samples were found to contain trichothecenes.

### Confirmation of wild rye (*Elymus* species) and foxtail barley (*H. jubatum*) as possible hosts of FGSC by artificial inoculation

**Figure 5:**
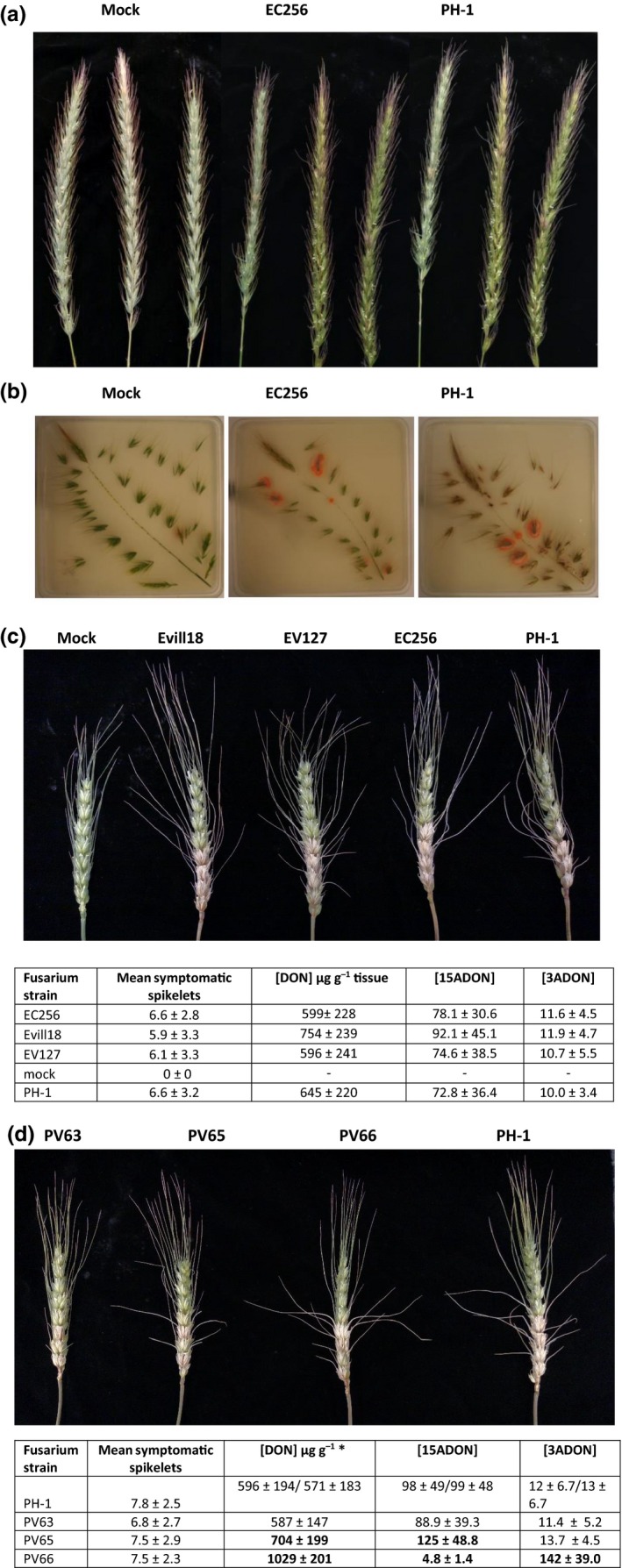
Infection of *Elymus canadensis* spikes with *Fusarium graminearum* derived from *E. canadensis* (EC256), with a Fusarium head blight (FHB) disease strain derived from wheat (PH‐1) or mock inoculated with water. (a) *E. canadensis* spikes, 14 d after inoculation, showing limited necrotic reaction towards EC256 (mean of 3.8 spikelets per spike) or PH‐1 (mean of 2.0 spikelets per spike) and non‐symptomatic, mock‐inoculated plants. (b) Dissected *E. canadensis* spikes plated on *Fusarium* selective medium. The salmon‐colored colonies are indicative of *F. graminearum*. Note *F. graminearum* colonies arise from spikelets inoculated with EC256 or PH‐1, and also occasionally arise from mock‐inoculated spikelets. On wheat (*Triticum aestivum* var. Norm), the virulence (mean symptomatic spikelets ± SD) of isolates from (c) *Elymus* species or (d) *Panicum virgatum* was not significantly different (*P *> 0.05) from the virulence of the wheat FHB strain PH‐1. Trichothecene levels accumulating on infection were not significantly different from PH‐1, except for strains PV65 and PV66. Trichothecene concentrations in bold are significantly different (*P *< 0.05) from values for PH‐1. The two trichothecene concentration values given for PH‐1 in (d) are for separate experiments comparing PV63 and PV65 (first PH‐1 values) or PV66 (second PH‐1 values).

All four plant species (*E. canadensis*,* E. villosus*,* E. virginicus* and *H. jubatum*) were successfully infected by each of the four FGSC strains based on the ability to re‐isolate *F. graminearum* cultures from the inoculated floret and, occasionally, florets distal to the point of inoculation. Unlike natural infection, some limited necrotic symptoms were found in response to artificial inoculation (Fig. 5a,b). Nevertheless, the ability of disease symptoms to spread within *Elymus* species was less than the ability of symptoms to spread within point‐inoculated wheat. All tested strains isolated from *Elymus* were as virulent on wheat as the authentic FHB strain PH‐1 (Fig. 5c). Little difference was noted among the *F. graminearum* strains used with respect to their limited ability to spread within *Elymus* plants. FGSC strains isolated from *P. virgatum* were also as virulent on wheat as PH‐1 (Fig. 5d).

In naturally infected *Elymus* species, as well as in *H. jubatum* tissue (from which strain HJ15 was recovered), trichothecene levels were below the limits of detection (Table S2). However, this was not the case for all grass species tested, as trichothecenes in trace amounts (< 0.28 μg g^−1^) were detected in 22 of 26 tissue samples from naturally infected *P. virgatum*, including the three samples from which strains PV63, PV65 and PV66 were isolated (Table S2; Fig. 5d). In artificially inoculated plants, low to moderate levels of trichothecenes (< 20 μg g^−1^) were detected in *E. canadensis* and *E. villosus* (Table 1). Even lower levels of DON (< 3 μg g^−1^) were detected in artificially inoculated *E. virginicus* and *H. jubatum*. These levels are 10–100‐fold lower than for similar inoculations of wheat (Fig. 5c,d), which ranged from 571 to 1029 μg g^−1^.

**Table 1:** Trichothecene accumulation in four grass species compared with accumulation in wheat

Fusarium strain	Host	DON (μg g^−1^)	15ADON (μg g^−1^)
EC256	*Elymus canadensis*	6.7 ± 2.7	2.6 ± 1.3
Noninoculated	*E. canadensis*	nd	nd
Mock	*E. canadensis*	nd	nd
PH‐1	*E. canadensis*	18.3 ± 12.5	7.5 ± 6.5
Evill18	*Elymus villosus*	10.2 ± 4.6	11.6 ± 4.9
Noninoculated	*E. villosus*	nd	nd
Mock	*E. villosus*	2.5 ± 5.1*	0.5 ± 0.9*
PH‐1	*E. villosus*	10.5 ± 1.9	10.2 ± 4.0
EV127	*Elymus virginicus*	2.9 ± 1.4	0.6 ± 0.2
Noninoculated	*E. virginicus*	nd	nd
Mock	*E. virginicus*	nd	nd
PH‐1	*E. virginicus*	nd	nd
HJ15	*Hordeum jubatum*	1.3 ± 0.5	0.5 ± 0.2
Mock	*H. jubatum*	nd	nd
PH‐1	*H. jubatum*	1.6 ± 0.7	1.0 ± 0.6
PH‐1	Wheat	276 ± 133	20.7 ± 10.3

Host plants were inoculated with a 15‐acetyl,deoxynivalenol (15ADON) chemotype strain derived from that host or a standard 15ADON strain used for Fusarium head blight (FHB) disease testing in wheat (PH‐1). Non‐inoculated plants or plants mock inoculated with the solution used to suspend spores were used as negative control. Mycotoxin values represent the mean concentrations (± SD) from four or more plant samples. Mean deoxynivalenol (DON) and 15ADON values for EC256 are significantly less than values for PH‐1 (*P *< 0.05). Note the accumulation of trichothecenes in mock‐inoculated *E. villosus* (*), indicative of pre‐existing *Fusarium graminearum* species complex (FGSC) infection. nd, not detected.

### Test for the presence of *Fusarium* in non‐inoculated or mock‐inoculated plants

*Fusarium* cultures were obtained from each non‐inoculated *Elymus* species. A FGSC strain was isolated from two of the six *E. villosus* plants tested. Two of the three seed‐grown *E. canadensis* plants contained *Fusarium* spp., including an FGSC strain from one plant and a *F. fujikuroi* species complex (FFSC) strain from the other. No *Fusarium* cultures were obtained from the three *E. canadensis* samples grown from plantlets. FGSC strains were obtained from two of six tested *E. virginicus* plants grown from seed. Wheat plants did not yield *Fusarium* outgrowth when the same tests were conducted for non‐inoculated plants grown from seed.

### Test for the presence of *F. graminearum* in seed of native grasses

Nine of the 26 grass species assayed harbored at least one *Fusarium* spp., and six plant species were shown to harbor FGSC strains. Of the nine grass species associated with at least one *Fusarium* species, three species (*L. oryzoides*,* E. villosus* and *E. hystrix*), from which FGSC strains were cultured, tested positive for the presence of DON, but only in trace amounts (< 0.25 ppm). Deoxynivalenol was not detected in bulk seed lots of the remaining three grass species from which FGSC strains were cultured (*B. latiglumis*,* G. striata* and *S. pectinata*). No DON was detected among the three grass species (*A. gerardii*,* Bromus ciliatus, K. macrantha*) where *Fusarium* isolates did not include FGSC strains. No trichothecenes other than DON were detected from any of the bulk grass seed lots. In total, 39 FGSC isolates were obtained from seed, originating from six native grass species (*B. latiglumis* (*n *= 7); *E. hystrix* (*n *= 5); *E. villosus* (*n *= 19); *G. striata* (*n *= 5); *L. oryzoides* (*n *= 1); *S. pectinata* (*n *= 2)). Genotyping for trichothecene chemotype revealed that all FGSC isolates were the 15ADON chemotype, except for a single strain, isolated from *B. latiglumis*, for which the genotypic profile matched the NX‐2 chemotype.

## Discussion

Here, we have shown that *F. graminearum* and related fungi in the *F. sambucinum* and *F. tricinctum* clades are prevalent, naturally occurring associates of native grasses in Minnesota. Further, we have shown that these *Fusarium* sp. are present in seed as well as foliar and floral tissues across phylogenetically diverse host lineages. Finally, we present what is, to our knowledge, the first report of trichothecene accumulation occurring in native, non‐cultivated grass species, naturally infected with FGSC strains.

Based on our sampling of FGSC strains arising from wild, non‐symptomatic plants, we infer that these *Fusarium* species are common endophytic fungi. Several lines of evidence support this inference. (1) Representatives of *Fusarium* species, previously recognized as plant endophytes, including FGSC, were recovered from plants growing in natural, non‐agricultural settings and from 17 of the 25 grass species sampled. More abundant and ubiquitous *Fusarium* species found in Minnesota prairie soil, such as *F. oxysporum* or *F. solani* (LeBlanc *et al*., 2017), were not recovered in the aboveground tissues sampled here. (2) FGSC strains were recovered from symptomless, non‐inoculated flowers of *Elymus* plants grown in the glasshouse. (3) FGSC strains were isolated from seed lots of *Elymus* and other native grass species. (4) Several bulked seed samples contained the FGSC mycotoxin DON. Collectively, these results suggest that FGSC strains are not merely quiescent residents of their hosts, but may be in an active metabolic state.

An interesting observation from this study was that spikelets from some mock‐inoculated *Elymus* plants showed traces of trichothecenes (Table 1). Although these *Elymus* plants may have been cross‐contaminated during the infection bioassay, similar tests of mock‐inoculated wheat plants have never yielded positive trichothecene levels in negative controls. Another possibility is that glasshouse‐grown *Elymus* plants naturally harbor endophytic FGSC strains, as we have described here from field‐collected samples. It is currently unknown exactly how native plants become infected with FGSC fungi but, as seed and non‐inoculated plants grown in the glasshouse contain FGSC infections, we speculate that these fungi may be vertically transmitted. To investigate whether seed lots contained visual evidence of fungal hyphae, *E. villosus* and *E. hystrix* seeds were surface sterilized, stained with Rose Bengal and separate tissue types were examined for the presence of fungal hyphae using a compound microscope. Both species were found to contain abundant fungal hyphae, lacking both clamp connections and spores, and extending into the inner seed coat (Fig. S1). Although it is not possible to determine fungal species using hyphal staining, and it is likely that multiple fungal species are endophytic in wild grass seed, taken collectively, we believe that this work provides a strong rationale for further investigation into whether *Fusarium* endophytes are vertically transmitted.

Naturally infected plants and seeds were non‐symptomatic and contained, at most, trace amounts of DON and related trichothecenes. On the other hand, infections resulting from point inoculation in the glasshouse (i.e. horizontal transmission) displayed limited necrotic symptoms and accumulated higher levels of trichothecenes. These two metrics of infection are notably related in cultivated plants, such as wheat and rice, where trichothecene accumulation is a known virulence factor associated with fungal spread and necrosis (Goswami & Kistler, 2005). Necrotic symptoms on wild grasses in the field were rare and, in this study, were never associated with *Fusarium* infection. Unlike the accumulation of trichothecene toxins in native grasses, infected wheat contained trichothecene concentrations that were orders of magnitude greater. The difference in trichothecene accumulation could be a result of the relative inability of native plants to induce trichothecene biosynthesis, their ability to actively suppress induction and/or their ability to metabolize trichothecenes so that they do not accumulate to high levels. Because associations between *Fusarium* and wild grasses appear to operate in a way that is fundamentally different from the associations between *Fusarium* and cultivated crops, future research to identify the mechanisms facilitating these interactions could prove valuable.

In environmentally collected plants from which FGSC cultures were isolated, 30% of the investigated grass species contained low levels of mycotoxins characteristic of the FGSC (DON, 15ADON, 3ADON, fusarenone‐X and zearalenone). The trichothecene chemotype frequencies of strains were similar to those found in strains isolated from wheat in the upper Midwestern USA (Kelly *et al*., 2015, 2016; Liang *et al*., 2015), except for a slightly higher occurrence of NX‐2 strains and the presence of NIV isolates. We also found that strains in the FGSC derived from non‐symptomatic native grasses are fully capable of causing FHB disease on wheat. An unanswered question raised by this study is whether the strains of the FGSC found in wild grasses are drawn from the same populations as those causing FHB disease epidemics of wheat and barley. Further population genetic analysis will be required to determine the relationship of FGSC populations on different hosts.

Collectively, our results highlight the broad extent to which *Fusarium* species infect wild grasses, including the potential of wild grasses to act as a reservoir for wheat‐infective FGSC strains. Because FGSC species divergence undoubtedly predated the development of cultivated crops, their interactions with wild grass species probably represent an evolutionary relationship involving the emergence of coexistence mechanisms that may have the potential for practical use. Elucidation of the mechanisms by which plants trigger or suppress trichothecene synthesis in the fungus, as well as detoxification mechanisms in the plant, will inform future crop development and breeding efforts of cultivated species currently impacted by FHB disease.

## Author contributions

C.W.K. and H.C.K. planned and designed the research. L.A.L., A.K.C., K.M.L., J.N., J.R., K.B., Y.D. and B.B. performed the experiments. N.R.L., B.B. and L.A.L. analyzed the data. L.A.L. and H.C.K. wrote the manuscript.

## Supporting information

Please note: Wiley Blackwell are not responsible for the content or functionality of any Supporting Information supplied by the authors. Any queries (other than missing material) should be directed to the *New Phytologist* Central Office.

**Fig. S1** Fungal hyphae growing within *Elymus* spp. seed coats: (a, b) *E. villosus*; (c) *E. hystrix*.
**Table S1** Plant species sampled for the presence of *Fusarium graminearum* species complex (FGSC) strains

**Table S2** Mycotoxin content of plant tissue naturally infected with *Fusarium*
