# Supplementary material for: Fusarium graminearum: pathogen or endophyte of North American grasses?
Source: New Phytol. 2017 Nov 21;217(3):1203–12. doi: 10.1111/nph.14894 (PMC5813145; doi:10.1111/nph.14894)
Supplement: Supplementary file 1 — Fig. S1 Fungal hyphae growing within Elymus spp. seed coats: (a, b) E. villosus; (c) E. hystrix. Table S1 Plant species sampled for the presence of Fusarium graminearum species complex (FGSC) strains [file NPH-217-1203-s001.pdf]

***Fusarium graminearum*: Pathogen or endophyte of North American grasses?**

Lotus A. Lofgren, Nicholas R. LeBlanc, Amanda K. Certano, Jonny Nachtigall, Kathryn M. LaBine, Jakob Riddle, Karen Broz, Yanhong Dong, Bianca Bethan, Christopher W. Kafer, H. Corby Kistler.

Acceptance date: 16 October 2017.

Plant Name	Common Name	Code	Isolates	Sequences	FGSC
Andropogon gerardii	Big Bluestem	AG	38	37	1
Bouteloua curtipendula	Sideoats Grama	BC	7	4	1
Bouteloua gracilis	Blue Grama	BG	17	16	2
Bromus ciliatus	Woodland Brome	BrC	49	41	5
Bromus kalmii	Prairie Brome	BrK	71	54	3
Bromus latiglumis	Earlyleaf Brome	BrL	3	0	0
Calamagrostis canadensis	Bluejoint	CC	20	18	2
Elymus canadensis	Canada Wildrye	EC	256	164	15
Elymus hystrix	Eastern Bottlebrush	EH	63	39	0
Elymus villosus	Hairy Wildrye	Evill	68	64	11
Elymus virginicus	Wild Rye	EV	254	182	11
Glyceria canadensis	Rattlesnake Mannagrass	GC	40	35	0
Glyceria grandis	American Mannagrass	GG	51	47	17
Glyceria striata	Fowl Mannagrass	GS	78	72	0
Hesperostipa spartea	Porcupinegrass	HS	54	28	0
Hordeum jubatum	Foxtail Barley	HJ	170	149	14
Koeleria macrantha	June Grass	KM	2	2	1
Leersia oryzoides	Rice Cutgrass	LO	6	4	3
Panicum virgatum	Switchgrass	PV	135	102	52
Phalaris arundinacea	Reed Canarygrass	PA	26	17	5
Poa pratensis	Kentucky Bluegrass	PP	1	1	0
Schizachyrium scoparium	Little Bluestem	SS	8	6	0
Sorghastrum nutans	Indiangrass	SN	5	4	0
Spartina pectinata	Cord Grass	SP	4	2	2
Sporobolus heterolepis	Prairie Dropseed	SH	9	8	1
	**TOTAL**		**1435**	**1096**	**146**

Table S1. Plant species sampled for the presence of *Fusarium graminearum* species complex (FGSC) strains. Listed are the binomial and common names of sampled plant species, their abbreviated code name, the number of fungal cultures established from each plant, the number of RPB2 DNA sequences obtained from isolated cultures and the number of cultures determined to be members of the FGSC from each host.

a)
b)
c)

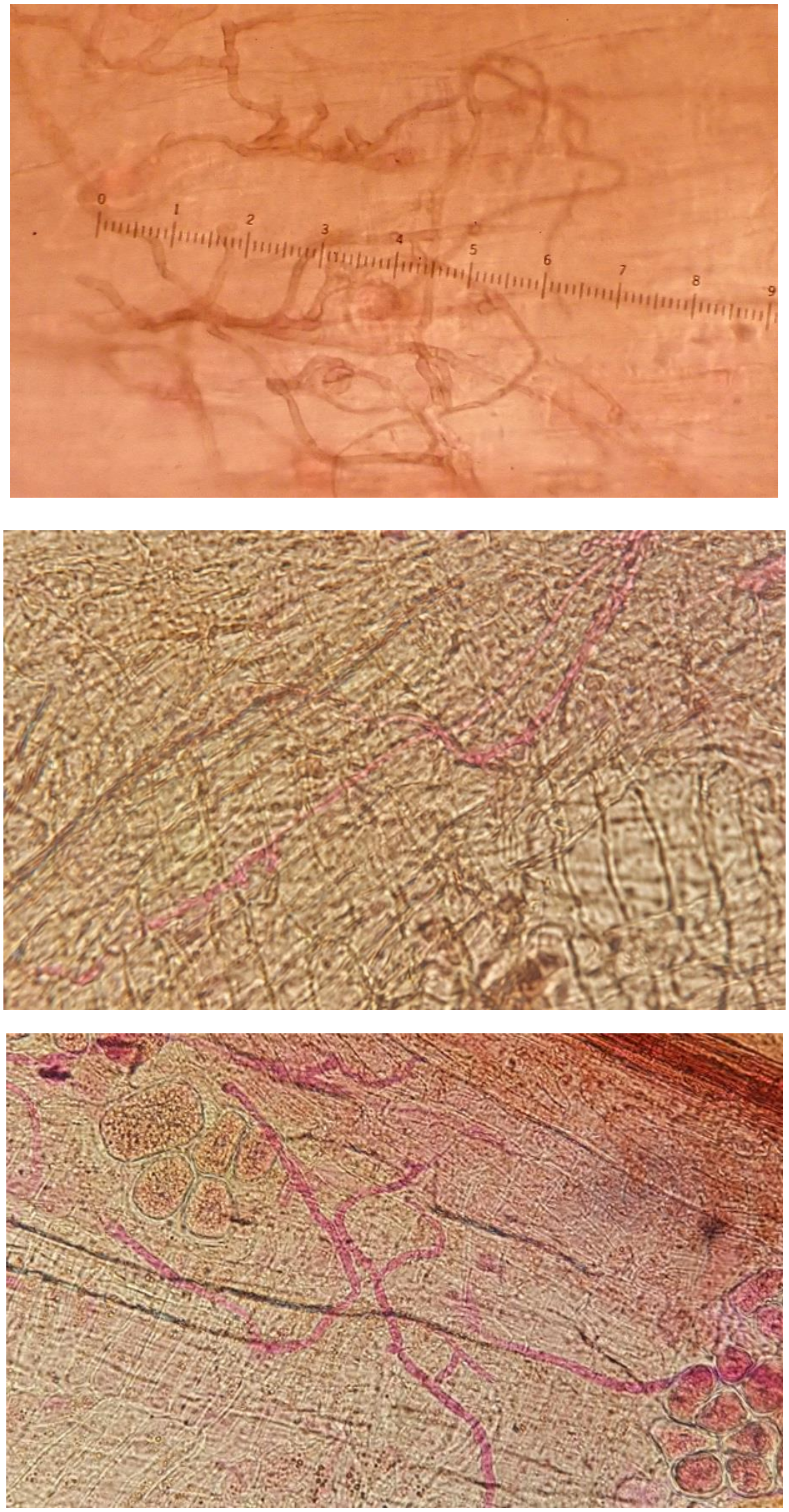

Figure S1. Fungal hyphae growing within *Elymus spp.* seed coats. (a)(b) *E. villosus*, (c) *E. hystrix*
